# University students’ perceptions of airborne infection control: exploratory study using Q methodology

**DOI:** 10.1186/s12889-020-09909-6

**Published:** 2021-01-04

**Authors:** Seonhye Lee, Hyun Jin Kim, Chang Heon Cheong

**Affiliations:** 1 Department of Nursing, Gyeongnam National University of Science and Technology, 33, Dongjin-ro, Jinju-si, Gyeongsangnam-do 52725 Republic of Korea; 2 Department of Nursing, Honam University, 120, Honamdae-gil, Gwangsan-gu, Gwangju, 62399 Republic of Korea; 3 Department of Architectural Engineering, Gyeongnam National University of Science and Technology, 33, Dongjin-ro, Jinju-si, Gyeongsangnam-do 52725 Republic of Korea

**Keywords:** Airborne infection, Pandemics, University student, Perception, Q methodology

## Abstract

**Background:**

International cooperation for infection control is important to prevent global pandemics. University students were difficult groups to manage of infection control measures. They often had overconfidence to their health, ineffective personal hygiene, and active social activities. Their misperceptions and inappropriate preventive behaviors increase the infection risks to university and community. Understanding university students’ perceptions of airborne infection management will contribute to the establishment of relevant policies and health education programs.

**Method:**

This study explored subjective perceptions of airborne infection among university students in South Korea using Q-methodology. Forty university students representing different majors ranked a set of 33 statements reflecting their perceptions of airborne infection. They sorted the statements into a distribution on seven scales ranging from “strongly disagree” to “strongly agree.” Collected data were analyzed by the PC-QUANL program. The subjective perception types were extracted by using the principal component analysis.

**Results:**

Four type are derived regardingperception of airborne infection: Type I (Government responsibility), Type II (Personal responsibility in self-management), Type III (Strict external management) and Type IV (Comprehensive countermeasures management). Thesefour types accounted for 45.6% of the total variance, and the individual contributions of Types I, II, III, and IV were 27.7, 7.6, 6.2, and 4.1%, respectively.

**Conclusion:**

The major contribution of this study is to clarify university students’ perceptions of airborne infection. These findings can be used in formulating effective strategies for health education, media reporting, and public health policy to improve airborne infection management.

**Supplementary Information:**

The online version contains supplementary material available at 10.1186/s12889-020-09909-6.

## Background

International cooperation for infection control is important to prevent global pandemics. In particular, rapidly spreading airborne diseases such as SARS (severe acute respiratory syndrome) and H1N1 (novel swine-origin influenza A) can cause increased casualties [1]. Airborne disease can be easily spread, creating outbreaks through loose infection management [2]. Respiratory infections are largely divided into droplet infection and airborne infection. Airborne infections occur when a person inhales airborne pathogens smaller than 5 μm floating in the air. A wide range of airborne infections is possible for the very small sizes and floating properties of airborne pathogens [1]. Therefore, infection management regarding building equipment operation such as HVAC (Heating, Ventilation and Air Conditioning) systems are strongly required to prevent the dispersion of airborne infections as well as personal infection control [3]. The combination of delayed recognition of the infectious condition, complex and dense hospital environments, uncontrolled movements of the infected patients, and delayed infection control interventions often cause serious spread of airborne infections [4]. Consequently, we need to perform affect-based assessment of risk and identify cognitive-based measures to prevent spread of airborne infection. These assessment and measures involve the preventing behaviors and adequate communication during pandemics [5]. In addition, personal beliefs and perceptions of airborne infections affect personal preventive actions [6]. Consequently, identifying public perceptions of airborne infections will help public education or policy-making about airborne infection management.

The South Korean government operates various campaigns and publicity to promote infection prevention activities and vaccinations. In particular, strengthening preventive education for young people with wide social relationships is important [7]. During the influenza outbreak, fewer than half of the respondent who studied in a university reported that they modified their behaviors to reduce their infection risk [8]. Nursing students also showed low perceived susceptibility when epidemics prevailed in their region [9]. Many students and faculties on campuses interact closely with surrounding communities, infection prevention activities at the university have a significant impact on community health care. Airborne infection prevention activities of college or university students have been studied in relation to knowledge, attitudes, and implementation [10, 11]. Individual awareness of the risk of infectious diseases is important to prevent the spread of epidemics [12]. Some studies using Q methodology show the perceptions of stakeholders such as disease management professionals [12, 13] and peoples in charge of vaccination [14, 15]. However, there are not enough studies using Q methodology to identify the public perceptions of airborne disease. Especially, college students do not sufficiently understand infectious diseases, but are convinced that they will not be infected because they are ‘fit and young’ [16]. Such misperceptions and inappropriate preventive behaviors increase the infection risk. Outbreaks of airborne disease often result inadverse effects, such as increased absenteeism, impaired school performance. Understanding university students’ perceptions of airborne infection management will contribute to the establishment of relevant policies and health education programs in university. University students have significant influences on the family, society and preliminary members of society. This study uses the Q methodology to identify the university students’ recognitions and their characteristics of airborne infection. The Q methodology is useful for representing a subjective perspectives (ie. feeling, emotion, attitude, perception, etc.) of a particular phenomenon and can be an appropriate method to understand the university students’ perceptions of airborne infection.

## Methods

### Design

This study is exploratory research using Q methodology to identify the structure of subjectivity that university students have about airborne infection.

### The Q-sample

The researchers derived the Q population (Q-set) through nonsystematic reviews and deep interviews. An active cooperation among governments, organizations and individualsare crucial factors for airborne infection management [17, 18]. The research team consists of school health and Q methodology experience (SL), student administrative manager (CHC), and mental health care and infection control specialists (HJK) established Q populations (Q-set) considering the relationships among individual, organization and government. The nonsystematic reviews are performed by scientific literature review and popular review. The scientific literature review used the database of pubMed, CINAHL (Cumulative Index to Nursing and Allied Health Literature), RISS (Research Information Sharing Service), KISS (Korean studies Information Service System), and NDSL (National Digital Science Library) of Korea. The popular reviews used the newspapers and internet articles. In-depth interview consists of five questions: fear or anxiety against airborne infection, preventive behavior promoter, barriers to preventive behavior, required efforts to prevent airborne infection in usual situation and required efforts to prevent airborne infection in pandemic situation. The In-depth interviews using the semi-structured questionnaire were conducted to four college students and four ordinary people within an hour by mental health care and infection control specialists (HJK).

According to the results of literature review and the in-depth interviews, 208 preliminary statements were derived. Each statement is integrated and briefly revised to exclude the similar or same meanings. The research team (SL, HJK, CHC) and three undergraduate students reviewed the validity and suitability of the statements. During content validity test, a statement with more than 80% of agreements are selected. We rechecked the suitability and understandability of the statements for the university students, and finally selected 33 Q statements for Q sorting. The statements are categorized at the individual level (fourteen statements), organizational level (seven statements) and governmental level (twelve statements).

### Participants

Forty university students(P-sample) interested in airborne infection agreed to participate in the interview. Students directly in the same department of the researcher were excluded. To reflect the various viewpoints of university students, students with various majors and grades are selected. Research participants are limited to those who understand the research purposes and agree to the research procedures.

Afterward, The socio-demographic characteristics of participants are acquired: grade, sex, age, major department, education related to infectious diseases, information sources about infectious disease and experience of infectious diseases in the past year (participants/their family).

The questionnaire for Q-classification is described in Additional file 1.

### Q-sorting

**Fig. 1:**
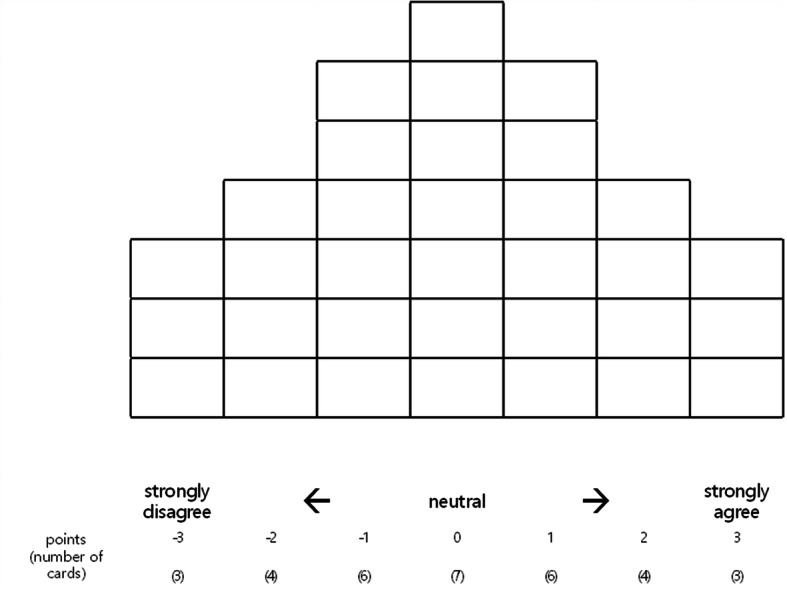
Q sort distribution

Q-sorting was performed in an open space such as cafeteria or study room. The researcher explained the purpose and procedure of the study to each interviewee. Afterward, a written consent for research participationwere made. The researcher explained prepared questions to each interviewee. The researcher placed individual statements one by one in each column of the Q-sorting distribution table according to the respondents’ perception. The order of Q-sorting was largely classified as disagreement, neutrality, and agreement, and then filled the cell from the most left side (− 3) and the most right side (+ 3) to the center of the Q-sorting distribution [19]. Each respondent placed three statements in the columns of − 3(strongly disagree) and + 3(strongly agree), four statements in − 2 and + 2, six statements in − 1 and + 1, and seven statements in the neutral column (0). Only one statement was allowed in one column (Fig. 1). Afterward each respondent sorted the statements to follow a quasi-normal distribution, that is, 3–4–6-7-6-4-3. Q-sorting was performed from December 14, 2018, to January 2, 2019. It took 30 to 45 min to complete the Q-sorting.

### Ethical considerations

This study was approved by the Public Institutional Bioethics Committee designated by the Ministry of Health and Welfare (Institutional review of board: IRB.P01–201809–23-007). Before interviews and data collecting, researchers explained to all respondents that all data acquired would not be used for any other purposes except forresearch and respondents could refuse an interview at any time. In addition, research procedures, guarantees for anonymity and privacy were explained to each respondent. Researchers explained to the in-depth interview participants about the voice recording procedure, storage, and destruction of recorded tapes. The Q-sorting was performed once the respondents wrote their written consent to participation.

### Data analysis

In the Q-sorting, highly disagree statements (− 3) were scored with 1 point. The neutral (0) and highly agree statements (+ 3) gain 4 points and 7 points respectively. The scores were coded in order of statement number and were analyzed by the PC-QUANL program. The PC-QUANAL program developed by Van Tubergen, a free software program using DOS program [19]. The types were extracted by using the principal component analysis [12–14]. Each typeof viewpoints represented a group of participants who ranked the Q statements similarly as statistically significant patterns [14, 19]. To determine the ideal number of types, the type of factor was analyzed based on Eigen value 1.0 or higher. The type of perception was analyzed based on the standard score (Z-score) of 33 Q statements for each type, the factor weight variance, and the general characteristics of the P sample.

## Results

**Fig. 2:**
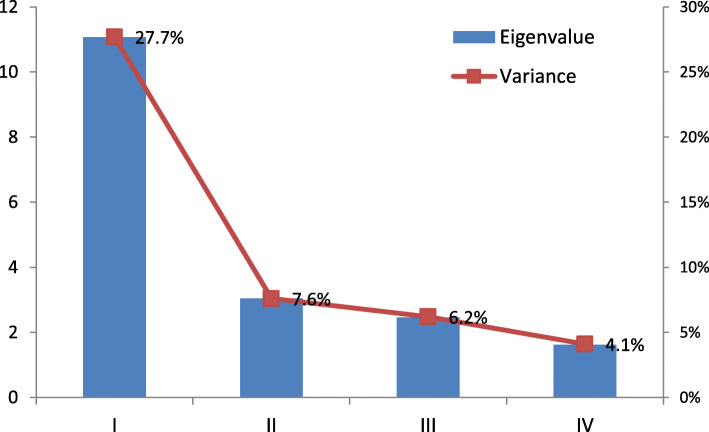
Eigenvalues and Variances according to types

**Fig. 3:**
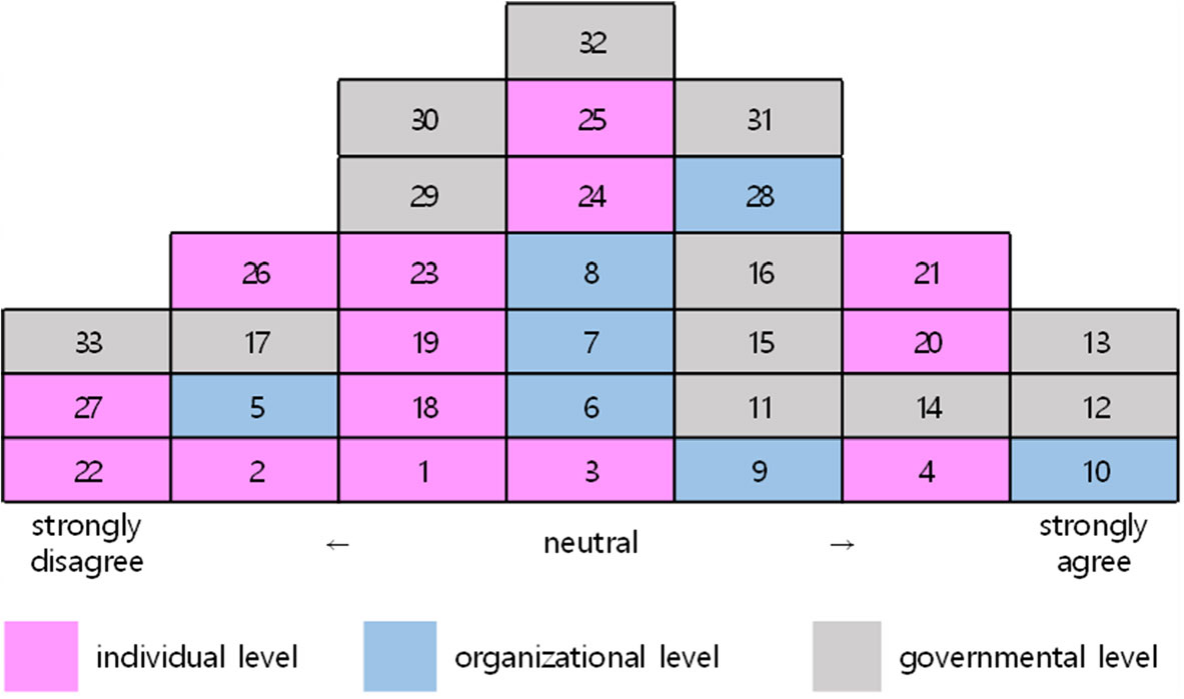
Type I: Government responsibility(#ID 12)

**Fig. 4:**
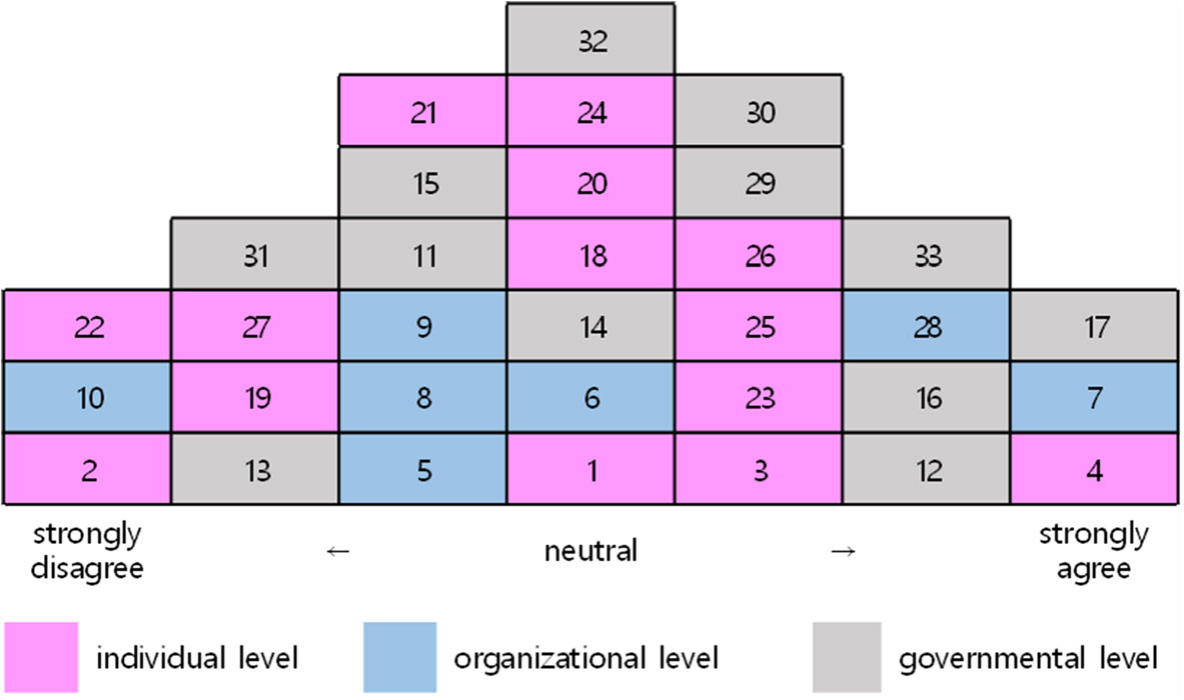
Type II: Personal responsibility in self-management(#ID 39)

**Fig. 5:**
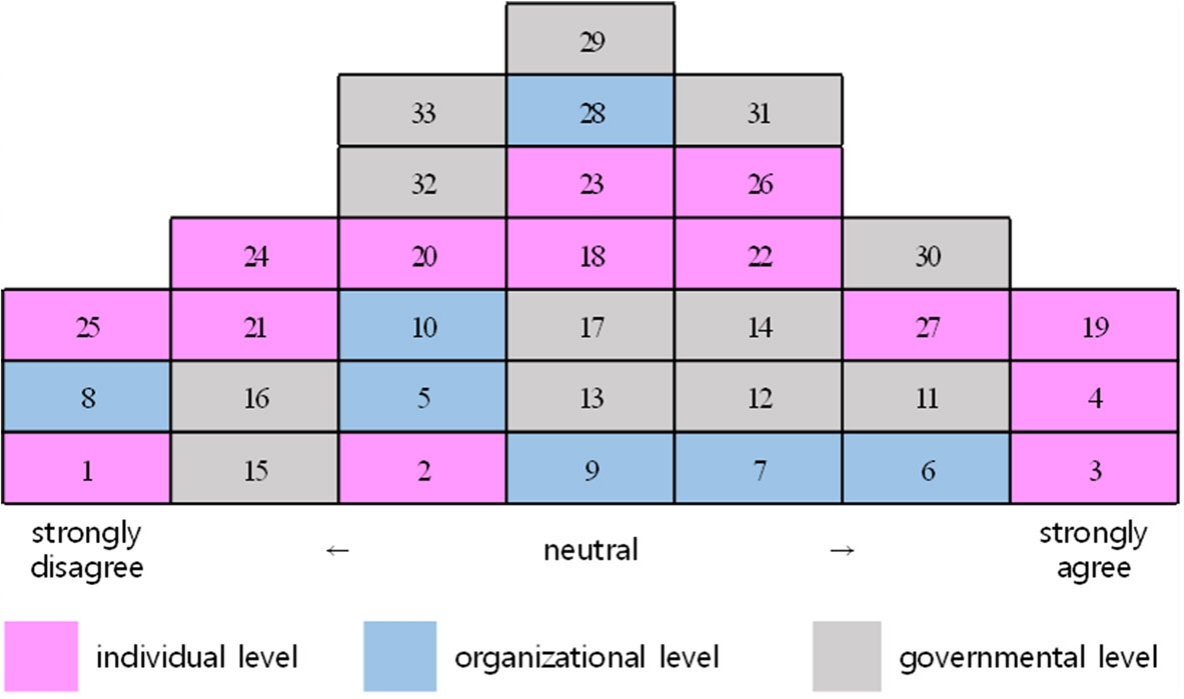
Type III: Strict external management(#ID 36)

**Fig. 6:**
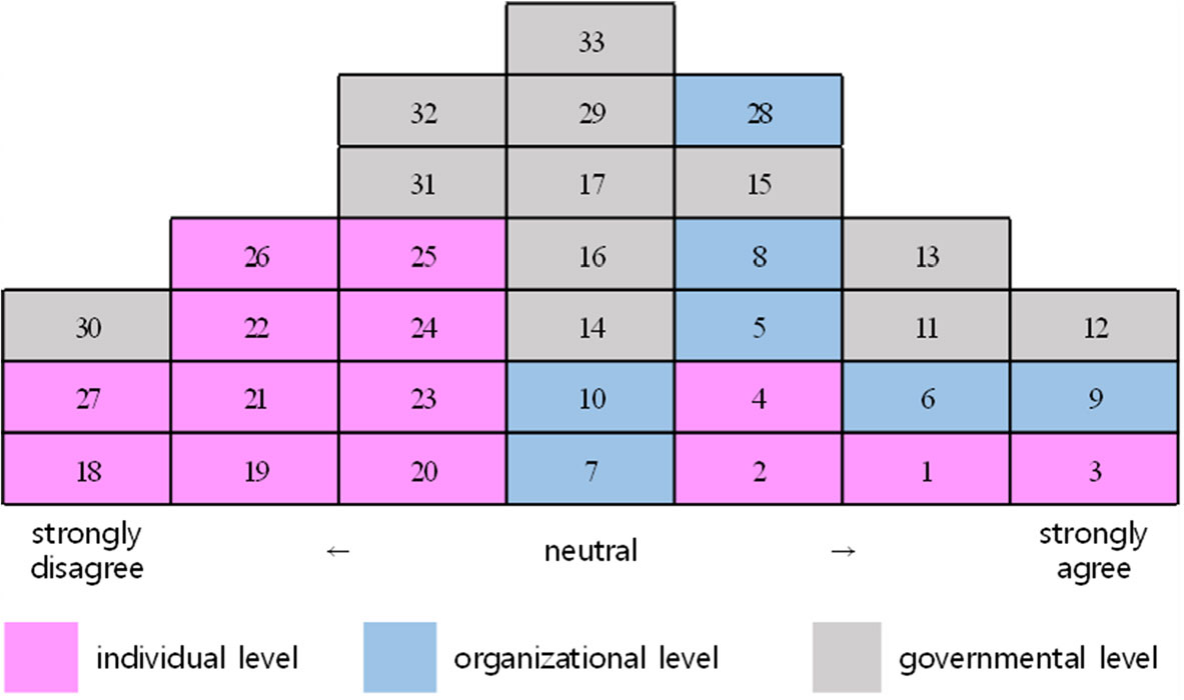
Type IV: Comprehensive countermeasures management(#ID 16)

Four types were derived as the representative perceptions of university students about airborne infection in South Korea. The explanatory powers for Types I, II, III, and IV were 27.7, 7.6, 6.2, and 4.1%, respectively. The explanatory power for all four types was 45.6% (Fig. 2). Q sort distributions of Type I, II, III, and IV were illustrated in Figs. 3, 4, 5, 6.

### Type I: government responsibility

Type I consist of twelve students (seven male students and five female students) with various grades, age and majors. Three students have received infection-related education. Their information sources on airborne infection were TV, the Internet, and medical staff, but some have “no source”. Interviewees in this group experienced five cases of individual infection and five cases of family infection (Table 1).

**Table 1:** Socio-demographic characteristics of the participants by types

Type	I (*n* = 12)		II (*n* = 8)		III *(n* = 11)		IV (*n* = 9)	
	NO.	(%)	NO.	(%)	NO.	(%)	NO.	(%)
**Grade**								
First	4	(33.3)	–	–	–	–	4	(44.4)
Second	5	(41.7)	3	(37.5)	5	(45.5)	1	(11.1)
Third	2	(16.7)	3	(37.5)	4	(36.4)	4	(44.4)
Fourth	1	(8.3)	2	(25.0)	1	(9.1)	–	–
**Sex**								
Male	7	(58.3)	6	(75.0)	6	(54.5)	4	(44.4)
Female	5	(41.7)	2	(25.0)	5	(45.5)	5	(55.6)
**Age group (yrs)**								
20–21	4	(33.3)	2	(25.0)	3	(27.3)	6	(66.7)
22–23	5	(41.7)	5	(62.5)	4	(36.4)	2	(22.2)
24–25	1	(8.3)	1	(12.5)	3	(27.3)	1	(11.1)
≥ 26	2	(16.7)	–	–	1	(9.1)	–	–
**Major department**								
Business & economics	3	(25.0)	–	–	4	(36.4)	1	(11.1)
Engineering	5	(41.7)	3	(37.5)	1	(9.1)	5	(55.6)
Humanities & social sciences	–	–	–	–	2	(18.2)	1	(11.1)
Nursing & public	1	(8.3)	–	–	1	(9.1)	–	–
Natural science	3	(25.0)	5	(62.5)	3	(27.3)	2	(22.2)
**Education related to infectious diseases**								
Yes	3	(25.0)	2	(25.0)	5	(45.5)	4	(44.4)
No	9	(75.0)	6	(75.0)	6	(54.5)	5	(55.6)
**Information source of infectious disease (multiple choice)**								
Television	5	(38.5)	2	(25.0)	5	(38.5)	2	(22.2)
Internet	5	(38.5)	6	(75.0)	3	(23.1)	6	(66.7)
Medical staff	1	(7.7)	–	–	2	(15.4)	–	–
No source	1	(7.7)	–	–	1	(7.7)	–	–
School	1	(7.7)	–	–	2	(15.4)	1	(11.1)
Social networking service	–	–	–	–	–	–	1	(11.1)
**Participant’s own experience of infectious diseases in the past year**								
Yes	5	(41.7)	4	(50.0)	5	(45.5)	3	(33.3)
No	7	(58.3)	4	(50.0)	6	(54.5)	6	(66.7)
**Experience of infectious diseases among participant’s family members in the past year**								
Yes	5	(41.7)	5	(62.5)	6	(54.5)	3	(33.3)
No	7	(58.3)	3	(37.5)	5	(45.5)	6	(66.7)

Type I emphasizes ‘preventive and management measures for airborne infection control by the national government (statement 14, Z= 2.05).’ Type I also claims that government should focus on the following statements: ‘more investment in preventive education or health promotion (statement 13, Z= 1.38),’ ‘reduction of inoculation prices, and expansion of free vaccinations (statement 12, Z= 1.30).’ Even though this type valued ‘citizens’ active cooperation on preventive measures (statement 11, Z= 1.26).’ On the other hand, Type I disagreeswith the following statements: ‘the resentful view that inappropriate management of patients were the cause of spread (statement 27, Z= -2.02)’, ‘the provision of much information through the media creates vague public anxieties (statement 33, Z= -1.94)’ (Table 2). Even though this type values the responsibilities and roles of the national government externally, also hasa vague fear of airborne infection internally. In addition, this type rarely performs individual prevention activities and has weak sense of responsibility. Type I generally shows strong consent instatements of organizational and governmental level (Fig. 3). Therefore, type I is named the “Government responsibility” regarding airborne infections.

**Table 2:** Q statements and *Z*-scores according to types

No	Q-statements (*: consensus statements)	*Z*-scores			
		Type I	Type II	Type III	Type IV
<Individual level>					
1	I am healthy and can recover quickly from an infection.	−0.91	1.90	− 1.49	0.78
2	I usually take preventive behaviors such as proper hand washing and wearing a mask.	−1.19	0.05	0.50	1.49
3	Hand washing, wearing a mask, and coughing manner are important to prevent infections.	0.74	1.78	1.28	1.55
4	An infected person should visit the hospital immediately to prevent the airborne infection.	1.18	1.51	2.23	1.78
18	During the pandemic, I am afraid to be with a person who is coughing or wearing a mask.	−0.59	−1.41	− 0.36	− 1.19
19	During the pandemic, I am afraid of being infected and infecting other family members.	−0.64	−0.95	1.19	−0.99
20	I have vague fears about airborne disease and no specific idea of prevention.	0.58	−0.23	−0.48	−0.58
21	Airborne infection cannot be prevented by personal efforts, and caught by bad luck.	−0.34	−1.56	−1.90	−0.49
22	I won’t go outside and I avoid crowded places during the pandemic.	−1.54	−1.16	−0.36	−1.36
23	I am not sure about the infection control and will be embarrassed a situation of infection.	−0.03	0.05	0.04	−1.25
24	I am afraid of people’s attention rather than of suffering from the infection.	−1.06	0.07	−1.12	−0.94
25	When the pandemic, those who perform thorough prevention measure are high sensitive.	−0.97	−0.55	−1.80	−0.31
26	The prevalence is the result of people who have noncompliance with personal measures.	−0.79	−0.19	−0.61	−0.28
27	I had a resentful view that inappropriate management of patients were the cause of spread.*	−2.02	−1.50	−1.27	−1.94
<Organizational level>					
5	My family is interested in health care and manages hygiene thoroughly.	−0.37	0.02	−0.57	0.81
6	Hospitals need simulation training to respond quickly such as pandemic infection.	0.71	0.06	1.06	1.03
7	Compliance and expansion of negative-pressure facilities are required for infection control.	0.32	1.06	0.05	0.80
8	Well-designed ventilation and operation system can prevent airborne infection.	−0.49	−0.19	−1.01	0.46
9	Schools should provide sufficient information and education about the airborne infections.	1.05	−0.41	0.77	1.05
10	It is urgent to improve the hospital visiting culture for the prevention of infection spread.	1.12	−1.29	−0.30	−0.18
28	The narrow aisle of beds and poor ventilation systems are major obstacles of prevention.	−0.22	0.46	−0.04	−0.61
<Governmental level>					
11	The citizen should actively cooperate with the measures of national and local governments.*	1.26	0.40	1.37	1.28
12	Government should lower vaccine prices and expand free vaccination.	1.30	2.11	0.90	1.47
13	Government should invest more in education/ promotion for the prevention of infection.	1.38	−1.50	0.00	−0.47
14	Government should make measures to prevent and manage airborne diseases.	2.05	0.13	0.35	0.63
15	Companies, schools, and the municipal government should supply masks for the citizens.	0.15	−0.94	0.03	0.32
16	A policy should be developed to respect the rights of infected person.	0.98	−0.12	0.39	0.33
17	To prevent pandemic, national inspection and isolation system should be set up.	0.81	1.33	1.04	0.63
29	There is a lack of public’s interest and education on the prevention.	−0.25	−0.23	0.64	−0.02
30	Anyone who doesn’t compliance of quarantine should be punish.	−0.48	0.12	1.13	−1.56
31	Government have a lack of policies and regulations to manage infection.	0.09	−0.43	0.48	−0.60
32	The domestic outbreak of infection is more problematic than overseas inflow.	0.09	1.20	−0.55	−1.10
33	It creates vague public anxieties that the media provide much information.	−1.94	0.42	−1.58	−0.52

### Type II: personal responsibility in self-management

Eight students (six male students, two female students) majoring engineering departments and natural science are belonged to Type II, but no first grade. Their ages were 20 to 23 years old. Two of them have received infection-related education. Their information sources about infection were TV and the Internet. Interviewees in this group experienced four cases of individual infection and five cases of family infection (Table 1).

Type II highly agrees with the statements: ‘take preventive behaviors such as proper hand washing and wearing a mask (statement 02, Z= 2.11)’, ‘health and quickly recovery from an infection (statement 01, Z= 1.90)’, ‘the importance of individual measure to airborne infection control (statement 03, Z = 1.78)’ and ‘quick visits to hospitals of infected persons (statement 04, Z= 1.51)’. The disagreed statements are ‘useless of preventive efforts and infected by bad luck (statement 21, Z= -1.56)’, ‘the resentful view that inappropriate management of patients were the cause of spread (statement 27, Z= -1.50)’, ‘more investment in preventive education or health promotion of government (statement 13, Z= -1.50)’, ‘a fear of mask-wearing and coughing person (statement 18, Z= -1.41)’, ‘improvement to the hospital visiting culture in South Korea for prevention to infection spread (statement 10, Z= -1.29)’ and ‘avoid to go outside and crowded places during the pandemic (statement 22, Z= -1.16)’ (Table 2).

Type II strongly emphasizes personal preventive activities and strongly agreed on the performance of strict self-preventive behaviors, fast recovery after infection, and the recognition of the importance of preventive activities. However, Type II has the low level of necessity for increased investments of education parts in the country and disease control to avoid infection during epidemics. Type II shows negative perceptions of the preparation of masks by organizations, abundant information provision and education by schools, improvement of hospital visiting culture, and increased investments in national education or promotion. Type II shows neutral attitude or consent in many statements of individual level (Fig. 4). Therefore, type II is termed as the “Personal responsibility in self-management.”

### Type III: strict external management

Type III have eleven students (six male students, five female). Type III has various ages and majorsbut no first grade. Five of them had received infection-related education. Their information sources about infection are diverse: TV, the Internet, medical staff, schools, and others. Interviewees in this group experienced five cases of individual infection and six cases of family infection (Table 1). The agreed statements of Type III are ‘quick visits to hospitals of infected persons (statement 04, Z= 2.23)’, ‘citizens’ active cooperation on preventive measures (statement 11, Z= 1.37)’, ‘the importance of individual measure to airborne infection control (statement 03, Z= 1.28)’, ‘be afraid of the infection or spreading to the family members when the pandemic (statement 19, Z= 1.19)’, ‘punishment to noncompliance of quarantine (statement 30, Z= 1.13)’, ‘the necessity of hospitals’ simulation training to respond at pandemic airborne infection (statement 06, Z = 1.06)’, ‘the necessity of national inspection and isolation system to prevent pandemic (statement 17, Z= 1.04)’, ‘well-designed ventilation and operation system can prevent airborne infection (statement 08, Z= -1.01)’, ‘fear of people’s attention rather than of suffering from the infection (statement 24, Z= -1.12)’, ‘the resentful view that inappropriate management of patients were the cause of spread (statement 27, Z= -1.27)’, ‘oneself health and quickly recovery from an infection (statement 01, Z= -1.49)’, ‘the provision of much information through the media creates vague public anxieties (statement 33, Z= -1.58)’, ‘people who had thorough compliance with prevention measure were high sensitive person (statement 25, Z =  - 1.80)’ and ‘useless of preventive efforts and infected by bad luck (statement 21, Z= -1.90)’ (Table 2).

Type III has positive perceptions of active cooperation with citizens, infection preventive activities, regularly emergency training in hospitals, and detection systems for infected travelers. Type III has a fear of infecting their families and demands severe punishment of non-cooperation with quarantine measures. Type III distrusts their own fast recovery from infections and preferred strict infection management. Type III shows strong consent in individual level statements. Unlike other types, Type III shows neutral attitude or consent at statements of organizational and governmental level (Fig. 5). Therefore, Type III is named the “Strict external management”.

### Type IV: comprehensive countermeasures management

Type IV have nine students of four males, five females but fourth grade. They areaged from 20 to 25 years old. Their majors are engineering, the natural sciences, the humanities, and commercial subjects (business and economics). Four of them have received infection-related education. Their information sources for airborne infection are TV, Internet, education at school and social network. Interviewees in this group experienced three cases of individual infection and three cases of family infection (Table 1).

The agreed statements for Type IV are ‘quick visits to hospitals of infected persons (statement 04, Z= 1.78)’, ‘the importance of individual measure to airborne infection control (statement 03, Z= 1.55)’, ‘take preventive behaviors such as proper hand washing and wearing a mask (statement 02, Z= 1.49)’, ‘reduction of inoculation prices, and expansion of free vaccinations (statement 12, Z= 1.47)’, ‘citizens’ active cooperation on preventive measures (statement 11, Z= 1.28)’, ‘provide to sufficient information and education about the airborne infections by school (statement 09, Z= 1.05)’ and ‘the necessity of hospitals’ simulation training to respond at pandemic airborne infection (statement 06, Z = 1.03)’. The disagreed statements are ‘the resentful view that inappropriate management of patients were the cause of spread (statement 27, Z= -1.94)’, ‘punishment to noncompliance of quarantine (statement 30, Z= -1.56)’, ‘avoid to go outside and crowded places during the pandemic (statement 22, Z= -1.36)’, ‘uncertainty to the management of airborne infections and embarrassment to pathogens’ exposure (statement 23, Z = − 1.25)’, ‘a fear of mask-wearing and coughing person (statement 18, Z= -1.19)’ and ‘domestic outbreak of infection is more problematic than overseas inflow (statement 32, Z= -1.10)’ (Table 2). This type is named the “Comprehensive countermeasures management” based on their evaluations of a comprehensive approach, including personal behaviors, family health management, prevention education in school, and the establishment of laws and management systems concerning airborne infection. Type IV balanced between positive and negative attitude toward statements of individual, organizational and governmental level (Fig. 6).

### The consensus statements

There are two concensus statements. The statement, ‘citizens’ active cooperation on preventive management activities (statement 11)’ obtained the most consent. On the other hand, the statement, ‘the view that inappropriate management of patients was the cause of epidemic infection (statement 27)’ gained the most opposition (Table 2).

## Discussion

This study identifies the perception types and their characteristics regarding airborne infection among university students using the Q methodology. Four types are derived regarding perception of airborne infection: government responsibility (type I), personal responsibility in self-management (type II), strict external management (type III), and comprehensive countermeasures management (type IV).

Types I and III emphasize external and government-centered management rather than individual management of airborne infection, accounting for 33.9% of the total variance. Type I focuses on improvement of hospital culture, benefits from precautionary action, necessity of education or promotion, but underestimated their own preventive behaviors. It is thought that these characteristic of type I resulted from the students’ insufficient knowledge and interest about airborne infection. In previous studies, students were not fully aware of the severity of infections, most of them had not learned any health-related behaviors. Only minority had leaned the simplest health-related behavior [11]. There were some skeptical college students questioned about the actual disease impact and thought that the government and the media only stressed “promptness” of preventive actions against airborne infection. A semi-structured interview with 20 participants showed that social distancing or social isolation was more difficult than hand-washing and mask wearing. Continuous exposure to infected individuals and disease transmission occurred during the pandemic, mainly through university club activities [16]. Therefore, universities should establish infection control policies in preparation for pandemics and encourage students to take preventive behaviors such as ongoing reminder, online health education, coughing measures, and provision of hand gel [11, 16].

Inappropriate information provision may cause unnecessary fear for some college students. Type III had negative feelings for the anxiety of providing information through the media, although there was a distrust of rapid recovery and a fear of airborne infection. They have a fear of family infections caused by themselves and a mistrust of their own rapid recovery from infection. These perception patterns are similar to the results of a prior study [20] that the worse the subjective health condition, the higher the worry. It seems that strict external measures to cope with infection are required due to a lack of self-confidence in health status or disease management. For this type, providing coping-oriented information that improves the self-efficacy of disease management will be more effective than providing negative health information, which increases fear [21]. In a precedent study [22], young students reported that information through the media, such as the Internet, TV, and radio, did not cause them anxiety. However, the level of the knowledge the young students learned from these media was low. The exposure to media alone could not accurately determine the absorption level of acquired information, and the amounts of information obtained were also largely dependent on the type of media [22]. The campaign for the young requires more diverse methods, such as social networks, information gateway gates, college e-mails, and newsletters [16]. University students are young and healthy, so they don’t modify their behaviors and have little interest in information acquisition [11, 16]. It is necessary to find out if educational messages cause vague fears of airborne infection in order to ensure that they could be effective channels of information delivery.

To these types, we need to pay more careful attention the students those are emphasizing government measures against infection and neglecting their own preventive actions. Under the attention of the university, students would be more independent, self-responsible for health management [23], and confident about managing airborne infection. Both national management and personal preventive actions are important for infection control, and participating in education strategies such as a forum or seminars seemed to be more effective than a unilateral health education. We will need to operate the health information sites in university. It will be a good channel to provide the supportive information for students’ health, enhance their health management and reduce vague fears or worries about infections.

Unlike the above two types, Type II values personal responsibility for self-management and included 7.6% of the participants. Type II distrusts the information provided by formal organizations such as schools and government institutions. They are also pessimistic about the risk of anxiety through the media and the effectiveness of investment to national public health education program. The students’ low confidence in formal organizations is manifested as high awareness of the importance of personal care due to the increased risk awareness of the disease. Although the low reliability of official organizations has a positive impact on individual management, trust is the basis for effective communication in the case of an outbreak [18]. Trust is also essential for disease management [17, 24] because it facilitates rapid response and reduces unnecessary behavior. This type requires a national approach of promotion to solve the issues of stigma and the insufficiency of individual approaches and personal preventive behaviors. It is indispensable for university students, who are part of a media-savvy generation, to develop e-health literacy competencies, and it is necessary to strengthen health communication through public health centers and Centers for Disease Control & Prevention.

The respondents of 4.1% think the management of comprehensive countermeasures to airborne infection was important. The public is not a passive entity anymore [25]. They had integrative views of airborne infection preventive measures at the individual-family-organization-governmental level: proper operation of ventilation systems, active cooperation among the people, the role of schools and hospitals, and the financial support of the state. Health is the most essential part of human life, and a common concept of interest among many college disciplines. They require personal management such as hand washing, mask wearing, and coughing etiquette. They also emphasize technological solutions, policy changes, and legal improvements such as air ventilation systems, negative pressure facilities, and protective equipment. For these various aspects and point of views, to prevent infection effectively, interdisciplinary research and education are strongly required. Recently, interdisciplinary education programs in universities have been developing. The related topics are worldwide problems such as climate change, environmental problems, an aging society and health, and international relations [26]. During an Ebola outbreak, Duke University nursing and engineering students developed a first-generation telerobotic intelligent nursing assistant to contact infected patients instead of healthcare providers at the site of infection [27].

Socio-demographic characteristics are different by the types. Students belong to type II and IVare well educated about infection. Type II obtains information on infectious diseases through TV and the internet. In previous study, 34.7% of university students answered the source of disease information through mass media (newspaper, TV) and the Internet [7]. Even when SARS were prevalent, the majority of students acquired basic knowledge of SARS and preventive health advice [16] through the internet [20]. It is necessary to identify the sources and contents of disease information through these media. The students’ perception of disease risk showed a negative correlation with the degree of trust in central administrative organizations and health policies [17]. Type IV collects information through school and social network service besides TV and the internet. There were more educated students with infection management than type I and type II. This type does not fear airborne infections and hold resentment against the infected situation. However, type IV usually practices preventive behaviors for themselves and their families. This type has high proportion of female students than other types. Precedent studies [17, 28] were similar with the findings of this study. Since the university students are collecting information from SNS and good at performing preventive management in usual, universities should provide practical information through SNS quickly. In addition, this type with a high percentage of male students showed a low attendance on infectious education, major with engineering or a natural, indicating a passive attitude towards managing airborne infectious diseases. Previous study [7] also explained that negative attitude of male students than female students, necessity of change in attitudes toward infectious diseases and a differentiated approach to health care management. To strengthen integrated management regarding infectious disease for university students, it is necessary to develop convergence and interdisciplinary projects.

According to the consensus statements, university students agree with accepting people’s responsibility while they disagree with actions that can cause stigma such as criticizing infected patients. Stigma may undermine the public health response to pandemics by strengthening barrier against acting to detection, treatment, and quarantine [29]. The students gave a positive perception that they value practice rather than stigma. In education programs for airborne infection prevention for students, a partnership approach that promotes cooperation will be more effective than a negative approach. It is necessary to hold a contest to stimulate academic curiosity about a wide range of infection countermeasures and to seek solutions for each major. To realize their new ideas, institutional arrangements and financial support are also needed. The university should create an environment that supports students’ personal hygiene and adheres to quarantine rules. Also, university should have the detailed measures to ensure the right to study and protect human rights for infected students.

The limitation of our study are the fact that it was involved the extraction of statements through a non-systematic literature review and used the small samples using non-randomization. This study is explorative rather than potential generalizable study. More research is needed to validate these patterns and viewpoints from this study. However, this study derived a basic understanding of university students’ perceptions of airborne infection. The results will contribute to establishing an effective education program for airborne infection prevention and mutual communication strategies.

We would like to propose further studies as follow. At first, it is necessary to identify variables affecting infection prevention behavior of each type. Secondly, educational programs to promote public health, improve self-management and encourage communication should be developed for each type. Thirdly, the factors influencing the university students’ self-efficacy and effective communication strategy related to airborne infection management should be identified. Finally, the research of perceptions’ type among the various country’s students should be prepared for pandemic outbreak in future.

## Conclusion

This study identifies the perception types of airborne infection among university students using the Q methodology and provids basic data to establish more effective infection control interventions in university. The study results show four types of perception of airborne infection that we have dubbed Type I (Government responsibility), Type II (Personal responsibility in self-management), Type III (Strict external management), Type IV (Comprehensive countermeasures management). These results will contribute to the development of health education program, media promotion, and health policy strategies for effective infection management in university.

## Supplementary Information

**Additional file 1.** Questionnaire for Q-classification.

## Data Availability

The datasets generated and analyzed during the current study are not publicly available due toparticipant’s privacy and decision of IRB but are available from the corresponding author on reasonable request.

## Abbreviations

CINAHL: Cumulative Index to Nursing and Allied Health Literature
H1N1: Novel swine-origin influenza A
HVAC: Heating, Ventilation and Air Conditioning
KISS: Korean studies Information Service System
NDSL: National Digital Science Library
RISS: Research Information Sharing Service
SARS: Severe acute respiratory syndrome
Z: Z-scores
