# Supplementary material for: University students’ perceptions of airborne infection control: exploratory study using Q methodology
Source: BMC Public Health. 2021 Jan 4;21:11. doi: 10.1186/s12889-020-09909-6 (PMC7781183; doi:10.1186/s12889-020-09909-6)
Supplement: Supplementary file 1 — Additional file 1. Questionnaire for Q-classification. [file 12889_2020_9909_MOESM1_ESM.docx]

**<Questionnaire for Q-classification>**

Thank you for sparing your precious time.

The purpose of this questionnaire is to identify ‘University students' perceptions of airborne infection control ', which is to read statements according to Q-methodological research procedure and arrange statements in the questionnaire according to agreement. The survey will be recorded separately and it will take 30 minutes to 1 hour. Therefore, please read all the statements and answer them straightforward. You can refuse to participate at any time if you are not willing to participate anymore.

All information regarding your personal profile in the survey will be kept anonymous and confidential, and will not be used for any purpose other than research. The Q-classification questionnaire will be kept together with the consent form for three years from the end of the study and will not be provided to third parties or other studies. When the storage period expires, we will destroy it using a document shredder.

Nothing is known about the risk factors associated with this research. Although the research does not directly benefit participants, your participation in this study will contribute to the development of policies and health education materials for the prevention of airborne infection.

Again, we sincerely thank you for your help in this study.

Researcher director : Seonhye Lee(010-5519-8520)

Co-researcher : Chang Heon Cheong(010-2621-6134)

Co-researcher : Hyun Jin Kim(010-2923-3192)

Here are questions about general characteristics. Please mark “V” where applicable.

1. Gender: ①Male ②Female
2. Age: ①Under 19(years) ②20-21(years) ③22-23(years) ④24-25(years) ⑤over 26(years)
3. Grade: ①Freshmen ②Sophomore ③Junior ④Senior
4. Major department: ①Humanities & Social science ②Business & economics ③Natural science ④Engineering ⑤Education ⑥Nursing & public ⑦etc. ( )
5. Experience in training related to infectious disease: ①Yes( ) ②No( )
6. Primary route to get information about infectious diseases: ①TV ②Internet ③Medical staff ④ etc.( )
7. Experience with infectious diseases in the past year: ①Yes( ) ②No( )
8. Experience of infectious diseases among family members in the past year: ①Yes( ) ②No( )

※ Please read following statements and arrange the statement number on the questionnaire according to your agreement.

1. I am healthy and can recover quickly from an infection.
2. I do preventive behaviors such as proper hand washing and mask wearing thoroughly to prevent infectious diseases.
3. Hand washing, wearing a mask, cough manner, vaccination, and strengthening immunity are important to prevent airborne infections.
4. An infected person should visit the hospital immediately to prevent the spread of airborne disease.
5. My family is interested in health care and manages hygiene thoroughly.
6. Under normal circumstances, hospitals need simulation training to respond quickly to emergencies such as large-scale air transmission infections or pandemic airborne diseases.
7. Hospital-related compliance and expansion of negative-pressure facilities are required for the prevention and control of airborne disease.
8. Well-designed ventilation systems and proper operation can prevent airborne infection in hospitals and schools.
9. Schools should provide sufficient information and education about the risks of airborne infections.
10. It is urgent to improve the hospital visiting culture in South Korea for the prevention of airborne infection spread.
11. The citizen should actively cooperate with the activities of national and local governments to prevent and control airborne disease.
12. To control the spread of airborne disease, government should lower vaccine prices and expand free vaccination.
13. Government should invest more in education and promotion for the prevention of airborne disease.
14. Government should make measures to prevent and manage airborne diseases.
15. Companies, schools, and the municipal government should supply masks for the citizens to prevent airborne transmission.
16. A policy should be developed to respect the rights of infected patients and the quarantine personnel.
17. To prevent the spread of pandemic airborne disease, national inspection and isolation system should be established for domestic and foreign travelers.
18. When airborne disease is prevalent, I am afraid to be with a person who is coughing or wearing a mask.
19. In the epidemic of airborne disease, I am afraid of being infected and infecting other family members.
20. I have vague fears about airborne disease and have no specific idea of prevention.
21. Airborne infection cannot be prevented by personal efforts, and caught by bad luck.
22. I won’t go outside and I avoid crowded places when airborne diseases are pandemic.
23. I am not sure about the nature and management method of airborne infections and will be embarrassed when exposed to airborne pathogens.
24. I am afraid of people’s attention rather than of suffering from an airborne disease.
25. When a pandemic airborne disease is prevalent, those who perform thorough epidemic prevention are overly sensitive.
26. The prevalence of airborne infections is the result of people who do not comply with personal preventive rules such as wearing masks.
27. The epidemic of airborne infections is a result of the patient’s poor management of the disease, so they are at fault.
28. The narrow aisle of beds and poor ventilation systems of hospitals are major obstacles to airborne disease prevention.
29. There is a lack of public interest and national education on the prevention of airborne disease.
30. Anyone who does not cooperate with a quarantine should be punished strictly.
31. There is a lack of government policies and regulations to manage airborne diseases.
32. The domestic outbreak and spread of airborne disease is more problematic than inflow from foreign countries.
33. Providing detailed and substantial information about airborne diseases through the mass media creates vague public anxieties.

※ Q-card classification distribution plot (e.g. 33 final statements)

- Please write down the corresponding number for each blank.


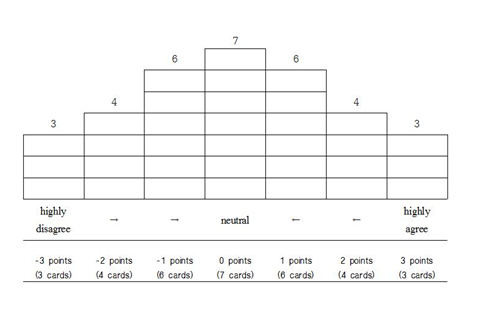


1. The numbers of three most disagreeable Q-statements, and reasons for selection.

| Q-statements’ number | Reason for selection |
| --- | --- |
|  |  |
|  |  |
|  |  |

1. The numbers of three most agreeable Q-statements, and reasons for the choice.

| Q-statements’ number | Reason for selection |
| --- | --- |
|  |  |
|  |  |
|  |  |
